# How does diabetes mellitus impact on the prognosis of dental implants?

**DOI:** 10.1038/s41432-023-00909-0

**Published:** 2023-08-10

**Authors:** Gabriele Baniulyte, Kamran Ali

**Affiliations:** 1 Oral and Maxillofacial Department, Royal Devon and Exeter Hospital, Barrack Road, Exeter, EX2 5DW UK; 2 Qatar University, Doha, 2713 Qatar

**Keywords:** Diseases, Oral diseases

## Abstract

**Data sources:**

Web of Science, Embase, PubMed and Cochrane Library databases were searched for publications up to August 2021.

**Study selection:**

The study noted clear inclusion and exclusion criteria. Search terms were provided; only observational studies were considered.

**Data extraction and synthesis:**

A total of 122 studies were identified through the search strategy. Following deduplication, two reviewers conducted the screening.

**Results:**

A total of 21 observational studies were included, involving cohort, case–control, and cross-sectional study designs. A meta-analysis identified increased risk of peri-implantitis in patients with diabetes mellitus and in smokers when compared to non-diabetic subjects and non-smokers. No significant association was found between poor plaque control or periodontal history and peri-implantitis.

**Conclusions:**

Patients with diabetes mellitus appear to have a higher risk of peri-implantitis.

## A Commentary on

**Li Y, Lu Z, Sun H**.

Impact of diabetes mellitus on the poor prognosis in patients with osseointegrated dental implants: a meta-analysis of observational studies. *Biotechnol Genet Eng Rev* 2023; 10.1080/02648725.2023.2184922.

**GRADE Rating:** 

## Commentary

Diabetes Mellitus (DM) is a complex metabolic disorder characterised by abnormalities in insulin secretion, absorption, or both, leading to significant health challenges^1^. If not effectively managed, it can cause persistent hyperglycaemia. This, in turn, can give rise to a multitude of complications, including microvascular disorders, compromised bone metabolism, increased vulnerability to infections, and delayed wound healing^1^. Therefore, it is important to assess and maintain optimal blood glucose control before undertaking invasive and potentially costly dental procedures, such as the placement of dental implants, to ensure their long-term success and durability.

The objective of this meta-analysis was to examine the association between DM and implant-related conditions with an impact on their long-term prognosis, including peri-implantitis and peri-implant mucositis. The authors conducted a search across four databases to identify relevant observational studies in English, involving patients with dental implants. The study population encompassed both individuals with DM and those with normal blood glucose levels (non-DM). Data collection and extraction was carried out by two independent reviewers, with the involvement of a third reviewer in case of any disagreements.

A total of 122 studies were identified. Following deduplication, ninety-six records were retrieved, thirty-five titles and abstracts were screened for eligibility and twenty-one studies were included in the meta-analysis. Among these, none of the studies were incorporated in the quantitative analysis to calculate a pooled Odds Ratio, capturing the relationship between DM and implant diseases. The combined dataset involved a cohort of 24,953 patients, with 1,526 individuals having DM and 23,427 individuals without DM. To assess any potential publication bias, an evaluation was performed utilising Begg’s test, revealing no evidence of publication bias across the included studies.

The meta-analysis involved several outcomes of interest. Nine studies were dedicated to examining the incidence of peri-implantitis between individuals with DM and non-DM, revealing a significant association between DM and peri-implantitis (*p* = 0.01). When compared to the non-DM group, the DM implant group displayed a 0.55-fold increased risk of developing peri-implantitis. Interestingly, the analysis did not uncover a heightened risk of peri-implant mucositis associated with DM (*p* = 0.34).

Furthermore, when investigating potential confounding factors related to peri-implantitis, smoking emerged as a significant risk factor (*p* < 0.001), increasing the likelihood of peri-implantitis by 1.754-fold compared to non-smokers. On the other hand, the analysis indicated that a history of periodontal disease was not significantly associated with peri-implantitis (*p* = 0.10). Similarly, plaque control did not demonstrate a significant correlation with peri-implantitis (*p* = 0.52).

While this meta-analysis was well-conducted, it is important to highlight several limitations. Firstly, the absence of a registered study protocol and the lack of adherence to PRISMA guidelines may raise concerns regarding the transparency and reproducibility of the study. Moreover, the search strategy did not incorporate grey literature or manual searching, potentially limiting the comprehensiveness of the review. Additionally, the inclusion criteria failed to provide a clear distinction between type 1 and type 2 DM. Consequently, the results did not differentiate between these two groups, which may have significant clinical implications. Furthermore, there was no explicit mention of the peri-implant disease classification employed in each study or whether it was determined through clinical or radiographic confirmation. Given the existence of various globally utilised classifications, this could introduce inconsistencies in the diagnosis of implant diseases, potentially impacting the comparability of results across studies. The study selection process also raises some concerns. Although 96 studies were identified after deduplication, the subsequent removal of studies was not adequately explained. Additionally, the number of studies that underwent full-text review and were ultimately excluded remains undisclosed. Furthermore, it is worth noting that out of the total participants included, only 1526 individuals had DM, compared to 23,427 non-DM patients. This significant difference in sample size may limit the generalisability of the findings, particularly for the DM group. Lastly, one of the reported results stated that peri-implantitis among non-smokers was associated with the risk of diabetes. However, this finding appears questionable, as it is well-established that multiple factors contribute to the development of diabetes, and it is unlikely that peri-implantitis alone would significantly influence diabetes risk. The abovementioned factors should be considered when interpreting the findings of this study.

The global burden of DM is staggering, with just under half a billion people worldwide currently living with this condition^2^. Projections indicate that this number is expected to rise by 25% by 2030 and surpass 50% by 2045^2^. These alarming statistics highlight the need to increase our understanding of diabetes and take proactive measures to mitigate the potential negative impact. It is important to note that DM prevalence is particularly high in urban communities and affluent nations^3^. These demographic factors should be considered, as dental implant procedures are frequently performed in urban populations with higher socioeconomic status. Furthermore, a significant concern is that half of all individuals living with diabetes remain undiagnosed^2^.

As demonstrated by this meta-analysis and other related studies, DM influences the long-term prognosis of dental implants. Notably, research has shown that diabetic patients who maintain good blood sugar control exhibit implant failure rates similar to those without diabetes^4,5^.

Therefore, the implementation of preoperative testing for glucose control could be suggested, ideally assessing and optimising the patient’s HbA1c levels, before proceeding with dental implant placement. This approach would not only facilitate the identification of patients with diabetes but also enable timely intervention to manage glucose levels effectively, consequently improving the overall survival and prognosis of dental procedures.

Practice points

- Sub-optimal glycaemic control in patients with diabetes may increases the risk of peri-implantitis.
- Smokers are also at a higher risk of peri-implant disease compared to non-smokers.
- Future studies should collect longitudinal data to quantify the risks of peri-implant disease in patients with diabetes mellitus.
